# Association of Genetic Variants of *BMP4* with Type 2 Diabetes Mellitus and Clinical Traits in a Chinese Han Population

**DOI:** 10.1155/2013/238150

**Published:** 2013-11-18

**Authors:** Shanshan Tang, Rong Zhang, Weihui Yu, Feng Jiang, Jie Wang, Miao Chen, Danfeng Peng, Jing Yan, Yuqian Bao, Weiping Jia

**Affiliations:** Department of Endocrinology and Metabolism, Shanghai Key Laboratory of Diabetes Mellitus, Shanghai Clinical Center for Diabetes, Shanghai Jiao Tong University Affiliated Sixth People's Hospital, 600 Yishan Road, Shanghai 200233, China

## Abstract

*BMP4* is one of the transforming growth factor-**β** superfamily, which can participate in adipogenesis. Gene encoding *BMP4* is acknowledged as a convincing candidate that may contribute to both glucose and lipid metabolism. In this paper, we aimed to test the impacts of *BMP4* variants on type 2 diabetes in a large sample of Chinese population. We genotyped 10 tagging single nucleotide polymorphisms within the *BMP4* region in 6822 participants and acquired detailed clinical investigations and biochemistry measurements. We found that *BMP4* rs8014363 showed nominal association towards type 2 diabetes, with the T allele conferring a high risk of type 2 diabetes (OR = 1.108, 95%CI 0.999–1.229, *P* = 0.051 for allele; OR = 1.110, 95%CI 1.000–1.231, *P* = 0.050 for genotype), but it was no longer statistically significant after adjusting for multiple testing (empirical *P* = 0.3689 for allele based on 10,000 permutations). Moreover, we observed a significant association of rs8014363 with triglyceride level and a trend towards association with high-density lipoprotein cholesterol after adjusting for age, gender, and BMI (*P* = 0.035 and 0.068, resp.). Our data suggested that the genetic variants of *BMP4* may not play a dominant role in glucose metabolism in Chinese Han population, but a minor effect cannot be ignored.

## 1. Background

According to the IDF Diabetes Atlas 2012, there are more than 371 million diabetic patients worldwide and 4.8 million patients died due to diabetes [1]. With the staggering increase of diabetes pandemic creating an overwhelming array of serious complications and high mortality rate, exploring the etiology behind diabetes is of great essentiality. Although environmental factors contribute significantly to diabetes, it is generally considered that the importance of genetic factors cannot be ignored as well [2, 3]. Recently along with the powerful genome-wide association study, the candidate gene approach can also guide a better understanding of the pathophysiology of complex diseases. Up to now, more than 60 loci have been confirmed to confer susceptibility to type 2 diabetes [4, 5] however, they are still not enough to interpret the genetic mechanism of the disease. As a consequence, it is worthy for further research.

Bone morphogenetic proteins (BMPs) belong to the transforming growth factor-*β* superfamily, which is now acknowledged to be involved in regulating embryonic development and differentiation as well as cellular function [6–8]. Among them, *BMP4* has been suggested to play an important role in adipogenesis, especially the white adipocyte differentiation through interaction with BMP receptor (BMPR) and subsequently activating the Smad signaling pathways [9–13]. White adipocyte tissues (WAT) are originally recognized as the primary site of triglycerides storage; however, accumulating evidences indicate that WAT is an endocrine organ that participates in the whole body energy metabolism and is highly associated with the risk of developing metabolic syndrome [14, 15]. Thus, *BMP4*, which is considered as a convincing candidate gene that may contribute substantially to both glucose and lipid metabolism, should arise more attention. Nevertheless, up to now, there is no report focusing on the genetic association studies of *BMP4* with type 2 diabetes and related clinical traits in East Asian population. In view of this, the aim of this current study was to test for the possible correlation between them in a Chinese Han population.

## 2. Methods

### 2.1. Ethics Statement

The study was approved by the institution review board of Shanghai Jiao Tong University Affiliated Sixth People's Hospital in accordance with the principle of the Helsinki Declaration II. Written informed consent was obtained from each participant.

### 2.2. Subjects

A total of 6822 participants of Han ancestry residing in Shanghai were recruited, including 3410 cases with type 2 diabetes and 3412 controls. Detailed information concerning this study population has been described elsewhere [16, 17]. In brief, all cases were unrelated type 2 diabetes patients defined according to 1999 WHO criteria and were recruited from the clinical inpatient database of Shanghai Diabetes Institute [18]. The controls were enrolled from community-based random sample epidemiological studies of diabetes and related metabolic diseases. All of them were unrelated subjects with normal glucose tolerance as assessed by 75 g oral glucose tolerant tests (OGTTs) and with negative family history of diabetes. The clinical characteristics of all participants were shown in Table 1.

### 2.3. Clinical Measurements

All participants underwent a detailed clinical investigation as described previously [19]. Briefly, anthropometric parameters such as height, weight, blood pressure, and waist and hip circumference were measured. Body mass index (BMI) was calculated as weight in kilometers divided by height in meters squared. For the controls, OGTTs which were assessed by standard 75 g glucose in the morning after an overnight fast were performed. And blood samples were obtained at the fasting and 2 h during OGTTs. Plasma glucose, serum insulin and lipid profile were measured. Homeostasis model assessment (HOMA), which was calculated by fasting plasma glucose and insulin, was used for estimating insulin resistance index and **β** cell function [20]. In addition, Insulin sensitivity from the OGTT was also estimated according to the insulin sensitivity index (ISI) proposed by Gutt et al. [21]. ISI =  [75000 + (fasting plasma glucose − 2 h plasma glucose) × 0.19 × weight]/120/mean plasma glucose/log_10_ mean insulin.

### 2.4. Single Nucleotide Polymorphism (SNP) Selection and Genotyping

In the present study, 10 tagging SNPs were selected according to the HapMap Phase III (release 27) Han Chinese database using the threshold of *r* ^2^ ≥ 0.8, which stretched 9 kb in the upstream to 9 kb in the downstream of the *BMP4* gene region. The 10 tagging SNPs could tag 73% SNPs (14 SNPs out of 19 SNPs in the HapMap Chinese Han sample) with a minor allele frequency (MAF) of >0.05. All the SNPs were genotyped using the primer extension of multiplex products with detection by matrix-assisted laser desorption ionization-time of flight mass spectroscopy using a MassARRAY Compact Analyzer (Sequenom, San Diego, CA, USA) and the overall call rate was 98.7%.

### 2.5. Statistical Analysis

The Hardy-Weinberg equilibrium test was performed in the cases and controls separately for each variant before association analysis. SNPs that failed this test (*P* < 0.01 in the controls) should be excluded. Pairwise linkage disequilibrium including |*D*′| and *r* ^2^ was estimated using Haploview (version 4.2). Allele and genotype distributions between the patients and control subjects were compared with *χ* ^2^ test or logistic regression [22], and odds ratios (ORs) with 95% confidence intervals (CIs) were presented. All skewly distributed quantitative traits (including fasting plasma glucose, 2 h plasma glucose, fasting insulin, 2 h insulin, triglycerides, total cholesterol, low-density lipoprotein cholesterol (LDL-C), high-density lipoprotein cholesterol (HDL-C), estimated ISI, HOMA for **β**-cell function, and insulin resistance) were logarithmically transformed to approximate univariate normality. Quantitative traits were analyzed in the control group by linear regression under an additive genetic model adjusted for age, gender, and BMI as confounding factors. Correction of multiple testing on allele association was performed using Haploview (version 4.2) through 10,000 permutations that randomly permutated the case/control status independently of genotypes. The statistical analyses were performed using SAS for Windows (version 8.0; SAS Institute, Cary, NC, USA). A two-tailed *P* value of 0.05 was considered statistically significant.

The statistic power was calculated under an additive model based on the allele frequency observed in our samples. Upon the assumption that the population risk was 9.6% and two-side *α* of 0.05, for SNPs with the minor allele frequency over 0.2, our case-control sample size has over 80% power to detect the minimum OR of 1.15.

## 3. Results

**Figure 1:**
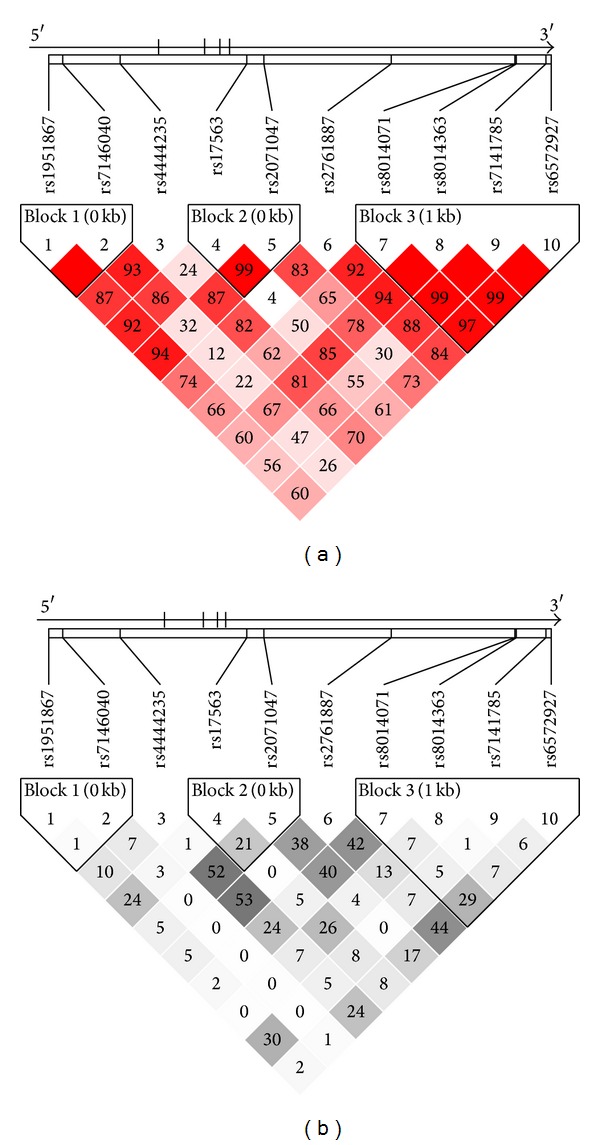
Linkage disequilibrium maps for SNPs genotyped in *BMP4* region. (a) Shades of red demonstrate the strength of the pairwise linkage disequilibrium based on *D*′ and numbers represent the value of *D*′ expressed as a percentage. (b) Shades of grey show the strength of the pairwise linkage disequilibrium based on *r* ^2^ and numbers indicate the value of *r* ^2^ expressed as a percentage.

The genotype distributions of all SNPs were in Hardy-Weinberg equilibrium. Pairwise linkage disequilibrium indicated that these 10 SNPs were in modest linkage disequilibrium and formed 3 haplotype blocks in this region (Figure 1).

We firstly examined the associations of these 10 SNPs with type 2 diabetes. Nominal evidence of the association was observed only for rs8014363, with the T allele conferring a high risk of type 2 diabetes (OR = 1.108, 95%CI 0.999 − 1.229, *P* = 0.051 for allele; OR = 1.110, 95%CI 1.000 − 1.231, *P* = 0.050 for genotype, Table 2). However, the result was no longer significant after correction for multiple testing by 10,000 permutations (empirical *P* = 0.3689). For the haplotype analysis, by comparing the frequencies between the cases and control subjects, we found that haplotype ACCT in block 3 comprised by rs8014071-rs8014363-rs7141785-rs6572927 indicated a marginal association with type 2 diabetes (*P* = 0.056, Table 3). After adjusting for multiple testing, the statistical significance was remarkably attenuated (empirical *P* = 0.4263).

In addition, we further analyzed the effect of the SNP rs8014363 on clinical characteristics in the control group under an additive model. As shown in Table 4, rs8014363 was significantly associated with triglyceride level, with carriers of more type 2 diabetes risk alleles (T) exhibiting higher values of triglyceride than CC homozygotes after adjusting for age, gender and BMI as confounding variants (*P* = 0.035). Moreover, we also observed a trend towards association between rs8014363 and HDL-C, with carriers of the risk alleles (T) demonstrating lower values (*P* = 0.068). However, no conspicuous associations were detected in other lipid profile indexes. 

## 4. Discussion

In the current study, we tested the effects of 10 tagging SNPs in *BMP4* region on type 2 diabetes in a Chinese population. We identified modest effects of *BMP4* variants on the risk of type 2 diabetes.

Over the past years, it has become increasingly obvious that obesity is a major independent risk factor for developing type 2 diabetes [23, 24]. In accompany with obesity, there is always increase of adipose tissues results from both adipocyte hypertrophy and hyperplasia [25]. *BMP4* is considered generally to induce commitment of pluripotent stem cells to the white adipocyte lineage [9–11]. In our present analysis, we observed that the triglyceride level significantly increased with the increasing number of risk allele in rs8014363. Nonetheless, an opposite situation between HDL-C levels and the number of risk allele in rs8014363 could also be seen. They are consistent with that WAT is a depot which is not only specialized in storing energy in the form of triglyceride but also a crucial target organ for insulin action. While HDL-C is mainly responsible for reverse-transporting cholesterol to the liver, it serves as a beneficial effect toward atherosclerosis, which is in contrast with triglyceride. That is to say, *BMP4* pose an influence on the glucose and lipid metabolism, which may through participating in WAT differentiation. 

Furthermore, One functional analysis using multiple genetic approaches showed that *BMP4* and its affinity BMPR1A were both expressed in *β* cells; besides, results not only in vitro but also in vivo all provided consistent evidences that *BMP4* in *β* cells was required for insulin secretion and advantageous to ameliorate glucose tolerance through significantly stimulating glucose-stimulated insulin secretion (GSIS) [26]. However, in our analysis, we failed to find any association with *β* cell function for variants of *BMP4*. Accordingly, the exact mechanisms behind these results should be warranted for further investigation.

Some limitations should be considered in the present study. Firstly, as the genetic effect of *BMP4* variants on type 2 diabetes was mild, our samples may not have enough power to detect the association. Secondly, because the patients we enrolled were not newly diagnosed and they were treated with antidiabetic drugs and/or insulin. The effect of *BMP4* variants on type 2 diabetes which is definitely through impact on impaired insulin secretion or insulin sensitivity is rather ambiguous. Thirdly, the status of taking lipid lowering medication in the control subjects was not clear. Nonetheless, the condition is very rare. Finally, although we found modest correlation between *BMP4* variant and type 2 diabetes and related clinical characteristics, we did not perform a replicated research in another independent sample to confirm these results. Thus, it is imperative to further replicate the influence of the variant of *BMP4* on type 2 diabetes and metabolic traits in other Chinese samples.

## 5. Conclusion

Our data suggested that the genetic variants of *BMP4* may not play a dominant role in glucose metabolism in Chinese Han population, but a minor effect cannot be ignored. Further investigations are of great necessity to confirm our observations and elucidate the unequivocal mechanisms underlying such association.

## Figures and Tables

**Table 1:** Clinical characteristics of the study sample.

	Cases	Controls
Samples (*n*)	3410	3412
Male/female (*n*)	1812/1597	1364/2048
Age (years)	60.33 ± 12.49	51.41 ± 14.39
BMI (kg/m^2^)	24.20 (22.00, 26.60)	23.23 (21.27, 27.68)
Fasting plasma glucose (mmol/L)	12.78 (9.00, 16.00)	5.02 (4.70, 5.40)
2 h plasma glucose (mmol/L)	17.00 (13.00, 22.00)	5.42 (4.60, 6.30)
Total cholesterol (mmol/L)	4.70 (4.00, 5.50)	4.70 (4.04, 5.35)
Triglyceride (mmol/L)	1.49 (0.99, 2.18)	1.25 (0.87, 1.82)
HDL-C (mmol/L)	1.11 (0.94, 1.33)	1.33 (1.13, 1.51)
LDL-C (mmol/L)	2.97 (2.42, 3.57)	3.04 (2.49, 3.61)

Data are shown as *n* or median (interquartile range).

BMI: body mass index; HDL-C: high-density lipoprotein cholesterol; LDL-C: low-density lipoprotein cholesterol.

**Table 2:** Associations of *BMP4* SNPs with type 2 diabetes.

SNP	Chr. position (Build 37.3)	Major/minor allele	Risk allele	Cases (*n* = 3410)		Controls (*n* = 3412)		OR for risk allele (95% CI)	*P* value for risk allele	OR for genotype (95% CI)	*P* value for genotype (empirical *P* value)
				Risk allele frequencies	Genotype count 11/12/22^#^	Risk allele frequencies	Genotype count 11/12/22^#^				
rs1951867	54407192	G/C	G	0.903	2748/586/35	0.896	2673/617/36	1.073 (0.959, 1.201)	0.220	1.071 (0.957, 1.198)	0.233
rs7146040	54407920	A/G	G	0.097	2755/586/36	0.096	2768/572/38	1.017 (0.908, 1.140)	0.767	0.983 (0.878, 1.101)	0.769
rs4444235	54410919	T/C	C	0.459	996/167/717	0.452	1034/1593/716	1.026 (0.959, 1.098)	0.461	0.975 (0.912, 1.043)	0.465
rs17563	54417522	T/C	T	0.720	1800/1319/273	0.717	1733/1375/269	1.042 (0.967, 1.124)	0.280	1.042 (0.967, 1.123)	0.283
rs2071047	54418411	C/T	T	0.368	1380/1522/485	0.355	1428/1527/444	1.056 (0.985, 1.133)	0.126	0.948 (0.885, 1.016)	0.131
rs2761887	54425052	C/A	A	0.519	793/1675/920	0.511	795/1674/866	1.033 (0.965, 1.105)	0.348	0.968 (0.905, 1.036)	0.348
rs8014071	54431500	A/G	G	0.350	1426/1572/404	0.341	1462/1539/386	1.039 (0.968, 1.115)	0.290	0.962 (0.896, 1.033)	0.287
rs8014363	54431575	T/C	T	0.884	2617/690/45	0.873	2574/774/45	1.108 (0.999, 1.229)	**0.051**	1.110 (1.000, 1.231)	**0.050** (0.3689)
rs7141785	54433114	C/T	C	0.904	2740/572/36	0.902	2727/594/32	1.028 (0.916, 1.154)	0.639	1.022 (0.912, 1.146)	0.705
rs6572927	54433390	T/A	T	0.633	1345/1534/456	0.632	1327/1533/455	1.008 (0.940, 1.082)	0.816	1.008 (0.939, 1.081)	0.834

*P* values <0.1 were shown in bold.

The additive model was used in the association analyses between genotype and type 2 diabetes.

^#^11: major allele homozygotes; 12: heterozygotes; 22: minor allele homozygotes. Empirical *P* values are for the alleles based on 10,000 permutations.

**Table 3:** Associations of three haplotypes in* BMP4* region with type 2 diabetes.

Haplotype	Haplotype frequencies		*P* value (empirical *P* value)
	Cases	Controls	
Block1 (rs1951867-rs7146040)			
GA	0.805	0.801	0.522
CA	0.097	0.103	0.248
GG	0.097	0.096	0.755
Block2 (rs17563-rs2071047)			
TT	0.368	0.355	0.116
TC	0.358	0.361	0.688
CC	0.274	0.284	0.210
Block3 (rs8014071-rs8014363-rs7141785-rs6572927)			
ATCA	0.365	0.366	0.960
GTCT	0.348	0.338	0.223
ACCT	0.117	0.127	**0.056** (0.4263)
ATTT	0.096	0.098	0.705
ATCT	0.073	0.068	0.247

*P* values <0.1 were shown in bold.

Empirical *P* values are for the haplotypes based on 10,000 permutations.

**Table 4:** Association analyses of the rs8014363 genotype with clinical characteristics in the normal glucose tolerant group.

	CC (*n* = 45)	CT (*n* = 774)	TT (*n* = 2574)	*β*	SE	*P *	P*
Age (years)	48.33 ± 14.93	51.02 ± 14.44	51.58 ± 14.39	0.7892	0.5317	0.138	/
BMI (kg/m^2^)	22.98 (21.48, 24.65)	23.26 (21.12, 25.53)	23.23 (21.30, 25.51)	0.0003	0.0022	0.884	/
Fasting plasma glucose (mmol/L)	5.06 (4.60, 5.40)	5.00 (4.63, 5.39)	5.03 (4.70, 5.40)	0.0015	0.0017	0.371	0.462
2 h plasma glucose (mmol/L)	5.09 (4.50, 6.14)	5.44 (4.70, 6.30)	5.43 (4.60, 6.34)	0.0017	0.0035	0.637	0.888
Fasting insulin (mU/L)	5.41 (3.39, 6.52)	6.00 (4.23, 8.34)	6.23 (4.39, 8.72)	0.0181	0.0115	0.114	0.127
2 h insulin (mU/L)	29.36 (12.43, 51.29)	28.34 (14.07, 45.17)	27.81 (16.10, 47.10)	0.0182	0.0158	0.249	0.365
Total cholesterol (mmol/L)	4.75 (4.04, 5.25)	4.64 (4.01, 5.32)	4.70 (4.05, 5.35)	0.0034	0.0036	0.341	0.643
Triglyceride (mmol/L)	1.11 (0.74, 1.71)	1.24 (0.84, 1.82)	1.26 (0.88, 1.83)	0.0188	0.0087	**0.031**	**0.035**
HDL-C (mmol/L)	1.38 (1.19, 1.58)	1.34 (1.14, 1.52)	1.33 (1.12, 1.51)	−0.0054	0.0036	0.134	**0.068**
LDL-C (mmol/L)	3.00 (2.45, 3.62)	3.03 (2.45, 3.60)	3.04 (2.50, 3.62)	0.0031	0.0049	0.527	0.846
HOMA-IR	1.13 (0.79, 1.56)	1.27 (0.91, 1.89)	1.34 (0.93, 1.91)	0.0201	0.0119	**0.092**	0.104
HOMA-B	79.81 (47.82, 128.65)	89.25 (61.45, 137.90)	90.30 (62.92, 135.68)	0.0115	0.0128	0.369	0.382
Gutt-ISI	104.36 (83.45, 138.38)	100.24 (81.62, 131.43)	99.56 (80.97, 127.80)	−0.0095	0.0068	0.160	0.220

Data are shown as mean ± SD or median (interquartile range).

BMI: body mass index; HDL-C: high-density lipoprotein cholesterol; LDL-C: low-density lipoprotein cholesterol; HOMA-IR: homeostasis assessment model of insulin resistance; HOMA-B: homeostasis assessment model of *β*-cell function; Gutt-ISI: insulin sensitivity index proposed by Gutt.

*P* values <0.1 were shown in bold.

*Adjusted for age, gender, and BMI.
